# Time division multiplexing based multi-spectral semantic camera for LiDAR applications

**DOI:** 10.1038/s41598-024-62342-2

**Published:** 2024-05-20

**Authors:** Sehyeon Kim, Tae-In Jeong, San Kim, Eunji Choi, Eunju Yang, Munki Song, Tae Joong Eom, Chang-Seok Kim, Alexander Gliserin, Seungchul Kim

**Affiliations:** 1 Department of Cogno-Mechatronics Engineering, College of Nanoscience and Nanotechnology, Pusan National University, Busan, 46241 Republic of Korea; 2 Department of Optics and Mechatronics Engineering, College of Nanoscience and Nanotechnology, Pusan National University, Busan, 46241 Republic of Korea

**Keywords:** Time division multiplexing, LiDAR, Multi-spectral camera, Time of flight, Imaging and sensing, Near-infrared spectroscopy

## Abstract

The recent progress in the development of measurement systems for autonomous recognition had a substantial impact on emerging technology in numerous fields, especially robotics and automotive applications. In particular, time-of-flight (TOF) based light detection and ranging (LiDAR) systems enable to map the surrounding environmental information over long distances and with high accuracy. The combination of advanced LiDAR with an artificial intelligence platform allows enhanced object recognition and classification, which however still suffers from limitations of inaccuracy and misidentification. Recently, multi-spectral LiDAR systems have been employed to increase the object recognition performance by additionally providing material information in the short-wave infrared (SWIR) range where the reflection spectrum characteristics are typically very sensitive to material properties. However, previous multi-spectral LiDAR systems utilized band-pass filters or complex dispersive optical systems and even required multiple photodetectors, adding complexity and cost. In this work, we propose a time-division-multiplexing (TDM) based multi-spectral LiDAR system for semantic object inference by the simultaneous acquisition of spatial and spectral information. By utilizing the TDM method, we enable the simultaneous acquisition of spatial and spectral information as well as a TOF based distance map with minimized optical loss using only a single photodetector. Our LiDAR system utilizes nanosecond pulses of five different wavelengths in the SWIR range to acquire sufficient material information in addition to 3D spatial information. To demonstrate the recognition performance, we map the multi-spectral image from a human hand, a mannequin hand, a fabric gloved hand, a nitrile gloved hand, and a printed human hand onto an RGB-color encoded image, which clearly visualizes spectral differences as RGB color depending on the material while having a similar shape. Additionally, the classification performance of the multi-spectral image is demonstrated with a convolution neural network (CNN) model using the full multi-spectral data set. Our work presents a compact novel spectroscopic LiDAR system, which provides increased recognition performance and thus a great potential to improve safety and reliability in autonomous driving.

## Introduction

Autonomous recognition systems have made remarkable progress in recent years and have various applications including robotics^1–4^ and driving systems^5–8^. Autonomous driving solutions have employed camera^9–14^ or light detection and ranging (LiDAR) systems^15–19^ to acquire the surrounding environment information during driving, which provides crucial feedback for navigation and safety strategies such as routing and collision avoidance. State-of-the-art time-of-flight (TOF) based LiDAR systems allow measuring object distances over hundreds of meters with centimeter accuracy^20,21^. The utilization of artificial intelligence technology with LiDAR systems remarkably improves the safety and reliability of autonomous driving^19,22–24^; however, the object recognition and classification through shape information alone still suffers from the problem of inaccuracy and misidentification, such as ice on the road^25,26^ or distinguishing between real people and human shaped objects such as mannequins or 2D pictures.

Recently, multi-spectral LiDAR systems have been introduced to overcome this limitation in object recognition by providing additional material information based on spectroscopic imaging^27–32^. Especially the reflection spectrum in the short-wave infrared (SWIR) range (900–2500 nm wavelength) provides more comprehensive information of the material properties compared to visible-range spectroscopy or simple color imaging of the object^32–35^. SWIR range multi-spectral LiDAR systems have demonstrated enhanced identification and recognition capabilities by simultaneous acquisition of spatial and spectral information. However, most of the previous multi-spectral LiDAR systems have employed spectrally resolved detection methods by using band-pass filters or complex dispersive optical systems^31,36,37^ which have inherent limitations. As the number of sampled wavelengths increases, not only does the inevitable optical loss increase but also the required multiple photodetectors and spectral separation of the reflected light make the system complex and expensive.

Here, we demonstrate a novel time-division-multiplexing (TDM) based multi-spectral LiDAR system for semantic object inference by the simultaneous acquisition of spatial and spectral information. The employed TDM method, which implements spectroscopy by sampling pulses of different wavelengths in the time domain, not only eliminates the optical loss in dispersive spectroscopy but also provides a simple, compact and cost-effective system. By minimizing the time delay between the pulses of different wavelengths within a TDM burst, all pulses arrive at the same location during a scan, thereby collecting spectral information from the same spot on the object, which simplifies data processing for the object classification and allows maintaining a sufficient scan rate of the LiDAR system. Our TDM based multi-spectral LiDAR system utilizes nanosecond pulse lasers with five different wavelengths (980 nm, 1060 nm, 1310 nm, 1550 nm, and 1650 nm) in the SWIR range covering a bandwidth of 670 nm to acquire sufficient material-dependent differences in reflectance.

For the simultaneous spatial and spectral visualization of the object classification, we assigned individual colors to the spatial intensity maps for each wavelength based on an RGB color model with priority. This approach maps the SWIR spectral information onto RGB colors, allowing for straightforward visual distinction of material properties with the human eye based on color differences. For the proof-of-concept demonstration, we compared such RGB-color encoded images of a human hand, a mannequin hand, a fabric gloved hand, a nitrile gloved hand, and a printed human hand, having similar visible-light colors, but showing clear differences depending on the material. Additionally, a convolution neural network (CNN) framework was trained on the full five-wavelength multi-spectral data set to demonstrate the material classification performance. The validation result of the trained model shows that the multi-spectral image is clearly classified with high accuracy according to the material. Additionally, we demonstrate the distance mapping with our TDM based multi-spectral LiDAR system which shows about 10 cm of ranging accuracy. These results signify a high application potential for advanced autonomous vehicle systems.

## Results and discussion

### Principle of the time division multiplexing (TDM) based multi-spectral LiDAR system

**Figure 1:**
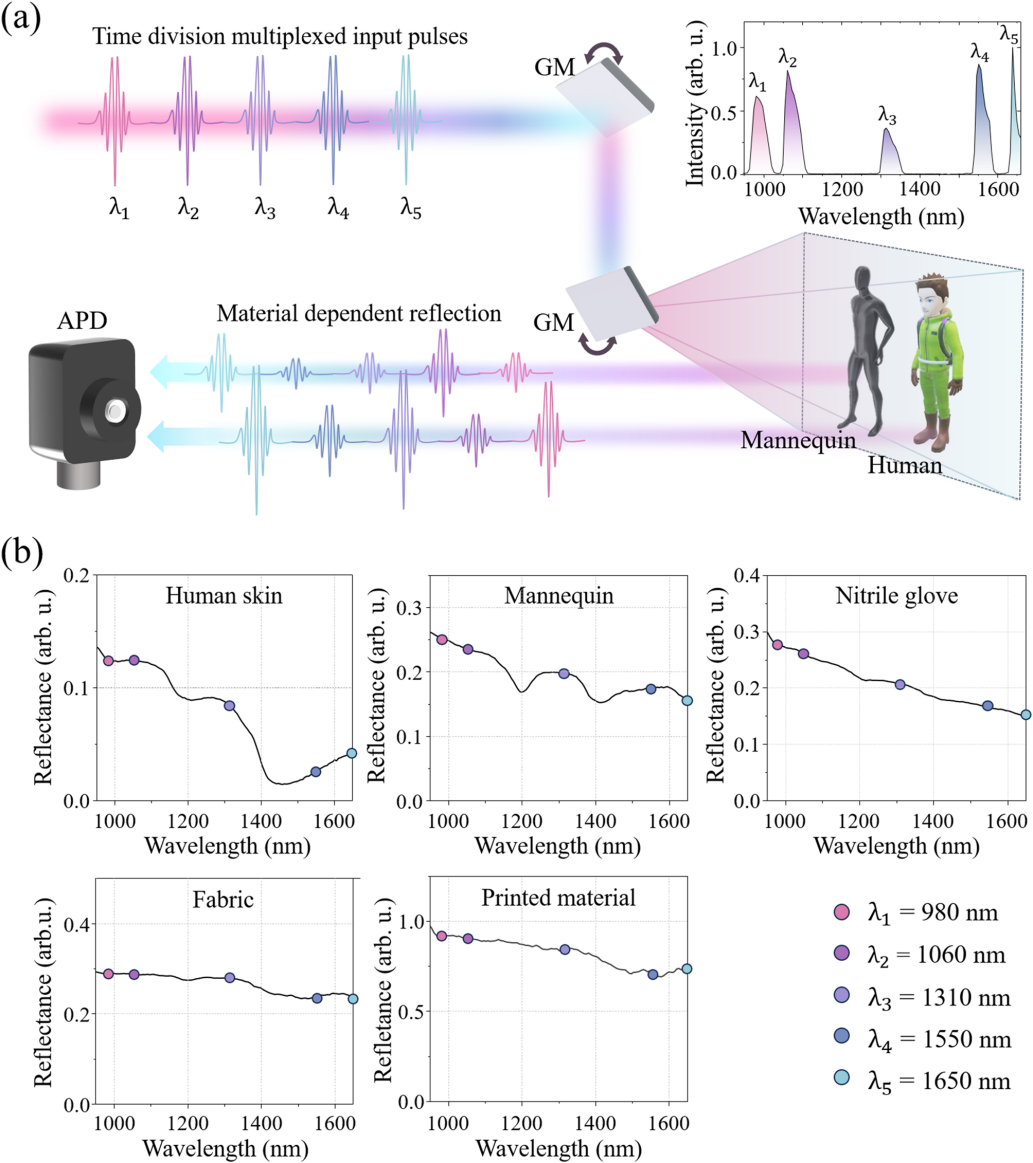
Principle of the TDM based multi-spectral semantic camera and object classification based on SWIR spectral fingerprinting. (**a**) Schematic illustration of the TDM based multi-spectral LiDAR system for target classification. The inset shows the spectrum of the five nanosecond pulsed laser sources in the SWIR range. GM: galvano mirror. (**b**) Reflection spectra of human skin, mannequin, nitrile glove, fabric, and printed material. The circles on the black solid lines represent the discrete wavelengths of 980 nm, 1060 nm, 1310 nm, 1550 nm, and 1650 nm of the five pulsed laser sources used in this work.

TDM is a ubiquitously used method in the field of data communication where multiple data streams are composed into a single signal by allocating different time slots within the signal relative to some data clock to carry short segments of the different data streams^38^. Through synchronization between the transmitter and receiver, TDM ensures accurate transmission and sampling of the individual data streams within the allocated time slots while using a single physical channel. A TDM based multi-spectral LiDAR system enables spectroscopy with a single spectrally integrating detector in the time domain unlike conventional spectroscopic LiDAR systems which rely on complicated spectrally resolved detectors^31,39^. The principle of our TDM based multi-spectral LiDAR system is illustrated in Fig. 1a. A collinear burst of five nanosecond laser pulses at different wavelengths in the SWIR range (Fig. 1a inset) with fixed time delays between the pulses is directed onto a galvano mirror to scan an object. The reflected burst of pulses is collected and focused onto a single avalanche photodetector to measure the distance via the time of flight as well as the amplitude ratio between the different pulses as a spectral fingerprint of the object, since the reflectivity in the SWIR range is usually highly sensitive to material properties. To demonstrate the potential of enhanced identification and recognition performance by using a multi-spectral LiDAR system, we chose a human as the object recognition target, since pedestrians constitute one of the most frequent hazards during driving. The SWIR reflection spectrum of the identification target (human skin) and relevant error objects (mannequin, fabric, printed human image, nitrile) were measured using a broadband tungsten-halogen light source (HL-2000, Ocean Optics) and a SWIR spectrometer (Flame-NIR, Ocean Optics) (Fig. 1b). The reflection spectrum shows clearly distinguishable differences depending on the material. In particular, the human skin is not only clearly distinguished from other objects but also exhibits a similar spectral response among numerous different test subjects, which is consistent with previous research^34^. This result shows that multi-spectral measurements in the SWIR range can provide sufficient information to classify the reflecting material.

### Multi-wavelength TDM synchronization, detection and image reconstruction

**Figure 2:**
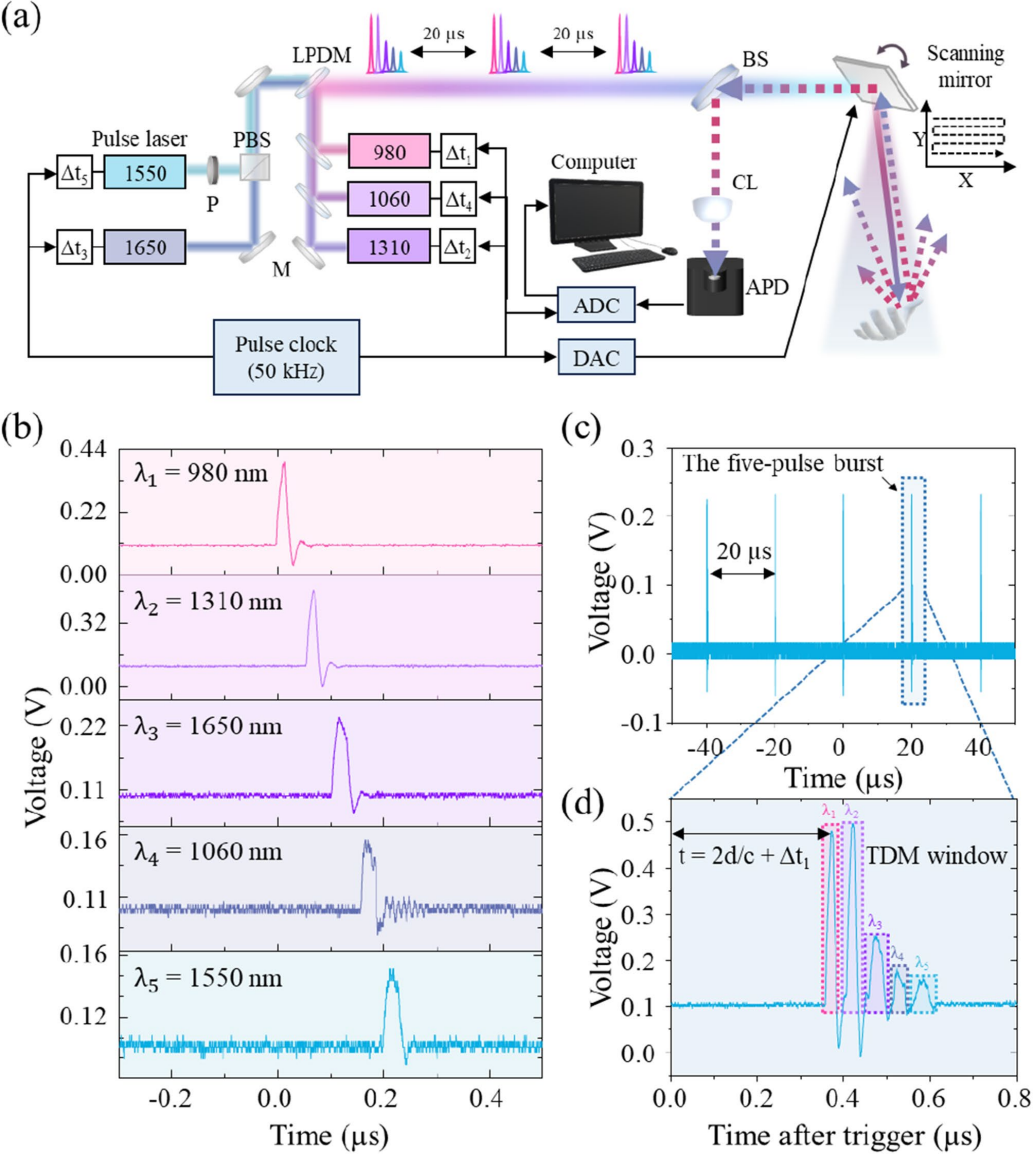
Operation principle of the TDM based multi-spectral LiDAR system. (**a**) Schematic illustration of the TDM based multi-spectral camera system. M: regular/dichroic mirrors; PBS: polarizing beam splitter; LPDM: long-pass dichroic mirror; BS: beam splitter; CL: collecting lens; APD: avalanche photodetector; ADC: analog–digital converter; DAC: digital-analog converter. (**b**) Delayed timing of the five individual nanosecond pulses constituting a burst. (**c**) Repetitive bursts of pulses with a 20 µs time interval generated by the pulse clock trigger. (**d**) Single burst of pulses with five individual integration windows yielding the five spectral intensities for this LiDAR image pixel. The TOF is determined by threshold or peak detection of the first laser pulse within the burst.

Five different commercial nanosecond pulsed diode laser sources (Aerodiode) at wavelengths of 980 nm, 1060 nm, 1310 nm, 1550 nm and 1650 nm, each with a bandwidth of approximately 40 nm (see Fig. 1a inset), are employed in the TDM based multi-spectral LiDAR camera system (Fig. 2a). The beams are collinearly combined in a home-built table-top setup using off-the-shelf optical components, such as individual dichroic mirrors for the three short-wavelength laser beams (DMLP series, Thorlabs) as well as a polarizing beam splitter (PBS, PBS254, Thorlabs) for the 1550 nm and 1650 nm lasers due to their higher efficiency, and the two resulting beams are combined with a long-pass dichroic mirror (LPDM, DMLP1500R, Thorlabs) to form a single beam in free space. The TDM multi-spectral measurement is controlled by a single clock source creating the required trigger signals to synchronize the pulsed laser sources, scanning, and data acquisition system. Here, we utilize a general-purpose data acquisition device (PCIe-6321, National Instruments), which provides programmable digital trigger capabilities as well as analog outputs for the beam scanning. The trigger pulses for the five lasers are derived from the pulse clock with an individual fixed nanosecond delay applied for each laser source (Δt_1_ − Δt_5_, Fig. 2b) resulting in a five-pulse burst for every clock pulse (Fig. 2c,d). The delays are chosen to bunch the individual pulses closely together in the time domain in order to maximize the available range for the time-of-flight measurement while avoiding temporal overlap between pulses. The bursts pass through a broad-band beam splitter (BS, BSW29R, Thorlabs) onto a gold mirror driven by a galvo scanner (SG2203, Sino-galvo) illuminating a spot on the object. The galvo scanner is driven by analog voltage signals for the X and Y axes controlling the deflection, which are provided by the built-in digital-to-analog converter (DAC) module of the data acquisition device used for trigger generation. The X axis of the galvo mirror oscillates rapidly back and forth with a triangular waveform synchronized with multiples of the clock pulses (defining the lateral resolution) while the Y axis moves slowly and continuously in one direction, creating a meandering scan over the object. Lastly, the back-scattered light is collected through the beam splitter and focused onto an avalanche photodetector (APD430C, Thorlabs) where the signal is recorded by a high-speed digitizer (ATS9371, Alazartech) at a rate of up to 1 GS/s, providing a 1-ns sampling resolution. Each pulse from the clock source triggers a continuous analog-to-digital conversion (ADC) of the photodetector signal over a fixed time window (shorter than the time between two trigger pulses) with the ADC data being buffered and continuously transferred to the computer such that no pulse is missed. The temporal position of the scattered burst within the ADC window constitutes a TOF measurement of the laser pulses to and from the object in addition to a fixed instrumental time offset (Fig. 2d).

The pulse clock corresponds to the pixel rate of the LiDAR imaging system and a trade-off has to be chosen between image resolution, image refresh rate, and maximum LiDAR range, while the temporal steepness of the laser pulse edge and the ADC sampling rate define the temporal and therefore distance resolution of the LiDAR system. In addition, the opening angle of the LiDAR camera is limited by the maximum mechanical deflection speed of the galvo scanner, which affects all other performance figures and limits our pulse clock to 50 kHz for reasonable measurement parameters, in particular image resolution (~ 40,000 pixels), refresh rate (~ 1 Hz) and opening angle (~ 50°). Apart from this mechanical limitation, the pulse clock speed is only limited by the shortest burst length achievable with the laser sources, which is around 300 ns for our five-pulse burst; shorter laser pulses are possible at the expense of a significantly reduced signal amplitude. Hence, the trigger system was designed for a maximum pulse clock of about 1 MHz, allowing for ~ 700 ns (or about 100 m) of usable LiDAR range. Even faster pulse clock speeds (and thus pixel rates) are in principle achievable at a reduced LiDAR range, since the nanosecond pulsed laser diode driver (CCS, Aerodiode) supports a repetition rate of up to 250 MHz. Since both the pixel clock and the image geometry are freely adjustable in our system, the ideal parameters can be chosen with priority on LiDAR range, pixel rate, or image refresh rate, depending on the particular application.

For best performance, the ADC data for an entire image scan, consisting of fixed-sized ADC records (one for each trigger pulse), is buffered on the computer and processed into a multi-spectral image stack while the next image is acquired in the background. Each ADC record is processed by first determining the temporal position of the burst, which provides the LiDAR TOF information. This can be achieved by a simple threshold detection of the first laser pulse’s edge within the burst, or by a more accurate peak detection algorithm at the expense of higher computation effort. Next, integration windows are applied to the five laser pulses within the burst using a fixed offset and length for each window relative to the measured temporal position of the burst (Fig. 2d). Each integration window is uniquely related to one particular laser source via the TDM method, yielding the five spectral intensities for this LiDAR image pixel. Since the sequence of the ADC records within the data set is related to the galvo mirror position during the image scan, the five integrated spectral intensity values in each ADC record can be easily composed into a multi-spectral image stack, i.e., a separate image for each laser wavelength, with additional depth information from the LiDAR TOF measurement. The processing of each ADC record can be done independently and in parallel for optimum performance.

### Visualization and semantic inference image processing of the multi-spectral data

**Figure 3:**
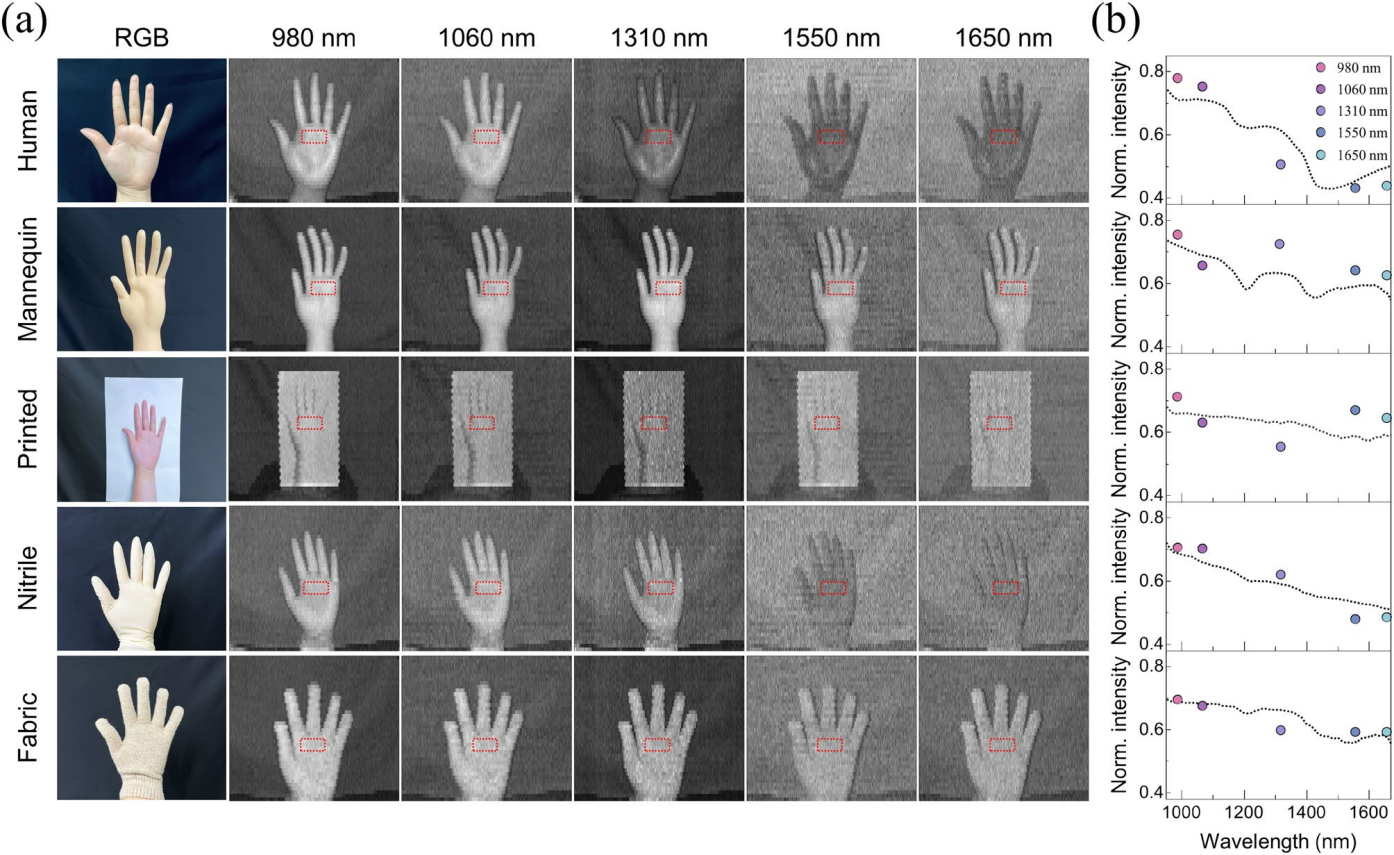
TDM multi-spectral imaging and spectroscopic analysis. (**a**) Gray-scale multi-spectral image stack for a human hand, a mannequin hand, a printed hand, a nitrile gloved hand, and a fabric gloved hand, as well as a color photograph for reference. (**b**) Normalized multi-spectral intensities in the red dotted region of interest in (**a**) for each object with the corresponding continuous reflection spectra of the target materials from Fig. 1b as a guide to the eye (black dotted lines).

**Figure 4:**
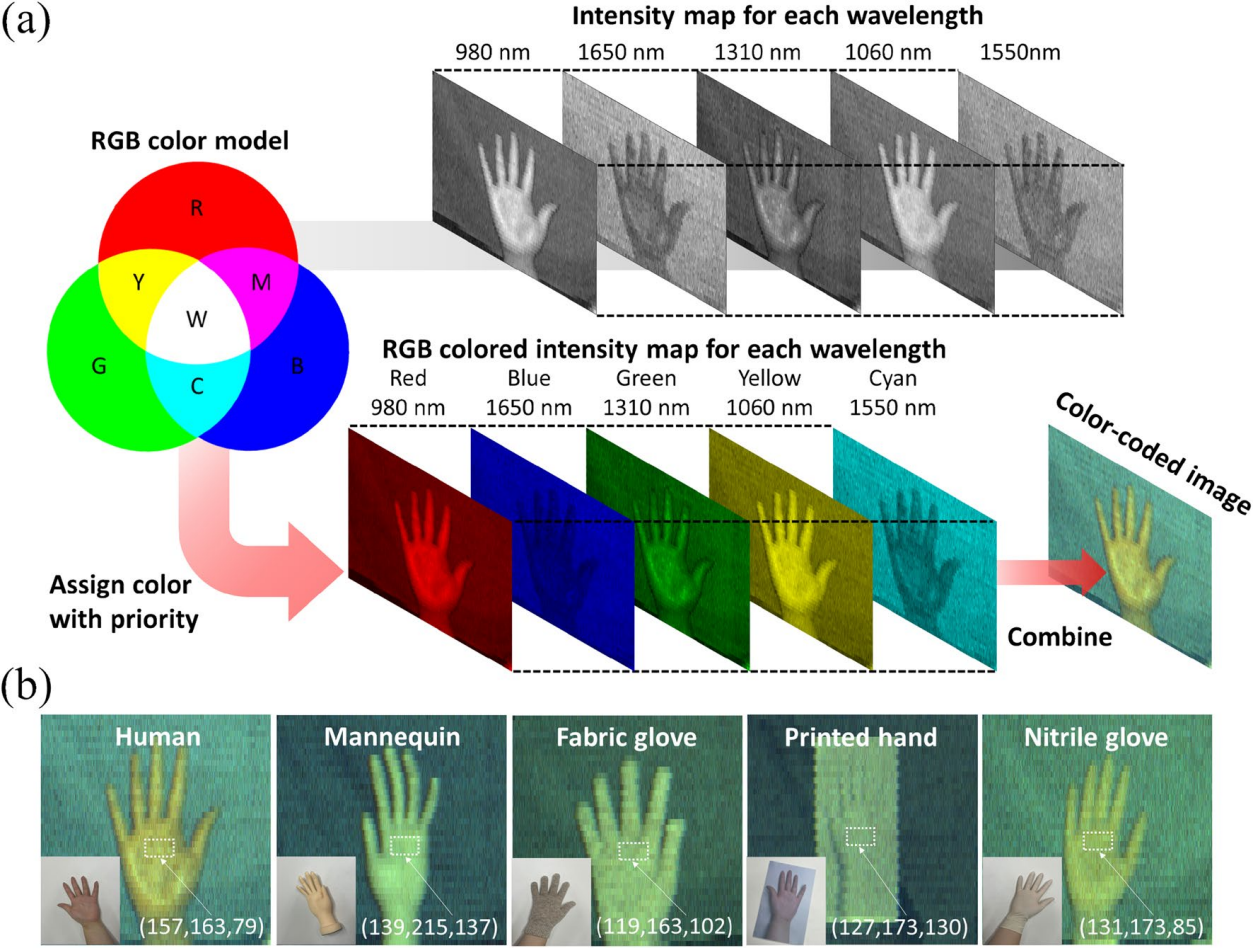
Multi-spectral visualization for various materials by RGB color-coding. (**a**) Principle of multi-spectral image processing for color coding. Primary and secondary colors of the RGB model are assigned to each wavelength with the primary colors given to wavelengths with the highest spectral variation between objects. R: red; G: green; B: blue; Y: yellow; C: cyan; M: magenta; W: white. (**b**) Multi-spectral RGB-color encoded images of various hand-shaped objects. The numerical RGB values refer to the white dotted region of interest.

After obtaining the individual images for each wavelength from the optimized parallel ADC record processing as gray-scale intensity maps, the material dependence of the SWIR reflection spectrum was demonstrated by using various hand-shaped target materials (human, mannequin, print, fabric glove, and nitrile glove) (Fig. 3a). For a quantitative analysis, the normalized spectral intensities at the individual wavelengths within the red dotted region of interest in Fig. 3a are shown as circles in Fig. 3b. The continuous SWIR reflection spectra from Fig. 1b obtained by a commercial spectrometer are plotted as black dotted lines as a guide and show a reasonable agreement, which demonstrates successful extraction of the relevant information for spectroscopic fingerprinting in the SWIR range by our method. The deviations are due to inconsistencies in measuring the spectral information at the exact same sample position as well as laser power drift. To distinguish the reflectance characteristic of different materials in the SWIR range with the human eye, we assigned an individual color to the intensity map for each of the five wavelengths based on the RGB color model. In this model, any color is represented by additive mixing of the three primary colors of light (red, green, and blue) expressed as R, G, and B values, which can be mixed to create secondary colors (yellow, cyan, and magenta). For a clear visualization, the three primary colors were assigned to the wavelengths with the strongest material-dependent variations (980 nm, 1310 nm and 1650 nm) and secondary colors to the auxiliary wavelengths (1060 nm, 1550 nm). The five colored intensity maps were then combined into an RGB-color encoded image, which allows distinguishing the material properties with the human eye (Fig. 4a). The visible-color encoded multi-spectral images for hand-shaped objects of various materials (human, mannequin, print, fabric glove, and nitrile glove) are shown in Fig. 4b along with the numerical RGB values (between 0 and 255) within the white dotted regions of interest, revealing significant material-dependent spectral differences encoded as RGB colors in a single image. Additionally, we studied differently colored cotton samples, which only differ by their color in the visible range but are made of the same material (Fig. S1). In this case, the image encoded in RGB color reveals no distinction between the objects, demonstrating that our method is capable of identifying different types of materials irrespective of their colors within the visible spectrum.

### Multi-spectral material species classification using a CNN model

The CNN architecture is most frequently employed as an artificial intelligence model for image classification because of its outstanding abilities to capture spatial patterns and hierarchical features in the visual data^24,40^. The 2D convolutional filter shares weights in the x and y dimensions to preserve spatial information and features while maintaining associations between neighboring pixels^41,42^. The excellent object recognition performance of CNNs makes them highly suitable to provide object recognition capabilities for autonomous vehicles, which are crucial for detecting potential obstacles such as pedestrians, other vehicles, and bad road conditions using a camera sensor^1,2^. However, a conventional camera image lacks the distinction between objects of different materials but of similar shape and color that frequently appear while driving. For example, it is difficult to distinguish between a pedestrian and a signboard depicting a human or between a wet and icy road surface^25,26^ using only a visible-light camera image. Here, we extend the superior shape recognition capabilities of a CNN with a multi-spectral signature in the SWIR wavelength range for enhanced autonomous object recognition using spectroscopic material information as an additional inference channel.

**Figure 5:**
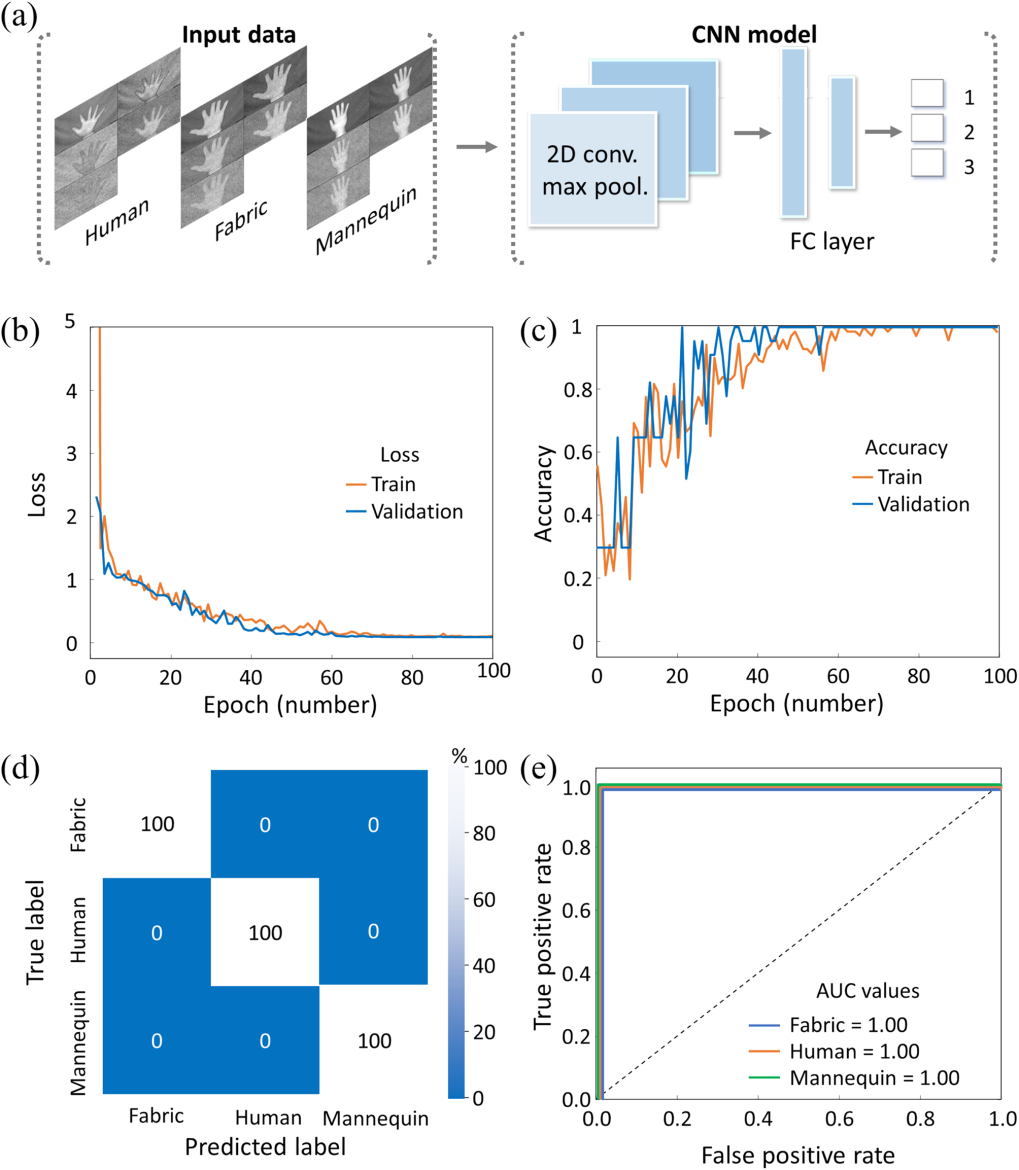
Material classification of multi-spectral images via the CNN platform. (**a**) Schematic of the CNN model training process with multi-spectral images from human, fabric, and mannequin hands. FC layer: fully connected layer. (**b**, **c**) Loss and accuracy values for each epoch for the training (orange lines) and validation (blue lines) data. (**d**) Confusion matrix values of the labeled and predicted material species. (**e)** ROC curves and their AUC values.

In order to apply our TDM multi-spectral imaging method to material species classification and evaluate its performance, we created a CNN model with multi-spectral images of a human hand, a fabric gloved hand, and a mannequin hand. The human hand and fabric gloved hand multi-spectral images were obtained from 40 different persons’ bare hands and wearing the same fabric glove, respectively, while 40 different multi-spectral images of the mannequin hand were acquired by repositioning the same mannequin hand. For the CNN model training and classification, the five-wavelength multi-spectral image stack was flattened by compositing the individual intensity maps into a single image (Fig. 5a) with fixed relative positions, thus retaining the full spatial and spectral information. The CNN model was designed with three filtering layers followed by a flattening layer with each building block comprising a series of 2D convolutional layers followed by a maximum pooling layer, and a rectified linear unit (ReLu) as an activation function. In particular, a softmax unit was employed as an activation function at the end of the fully connected layer to classify the material species (Fig. 5a). The adaptive moment estimation (Adam) method was employed as an optimizer, which is a stochastic gradient descent method based on adaptive estimation of the first and second moments. The loss and accuracy curves represent the learning process of the model, which is composed of a training part (orange lines) and a validation part (blue lines) (Fig. 5b,c). The loss function is the difference between the true value and the value predicted by the model, which is utilized to optimize the weight parameters in the neural network. The accuracy value indicates the performance of the model for the entire data set as the fraction of correct predictions, i.e., the ratio of predictions where the predicted value is equal to the true value. The loss and accuracy converge to 0 and 1, respectively, with increasing epoch number, and the training part shows a similar trend as the validation part, which indicates that the CNN model is optimized without overfitting issues.

A confusion matrix (Fig. 5d) represents the prediction distribution of the model for each actual label in the classification data set as a percentage. Here, the principal diagonal percentage indicates the accuracy of the model to correctly classify the data, and the result shows that the optimized CNN model can successfully classify the multi-spectral image based on material species. Furthermore, the classifier performance is summarized by plotting a receiver operating characteristic (ROC) curve (Fig. 5e), which compares the rate of true positives with the rate of false positives for our CNN classifier using the results of the confusion matrix (the dashed diagonal line represents a random-guess classifier for reference). The area under this curve (AUC) provides a normalized statistical metric for the performance of a CNN classifier, also referred to as an index of accuracy, where a perfect classifier has an AUC value of 1 (no false positives or negatives) and a random-guess classifier has an AUC value of 0.5. Our optimized CNN model classified all samples without error and thus achieved a perfect AUC value of 1. Although the data set was somewhat limited, these results demonstrate that our TDM based multi-spectral LiDAR system and data processing method are well suited for accurate semantic inference of the material species.

### Demonstration of light detection and ranging

**Figure 6:**
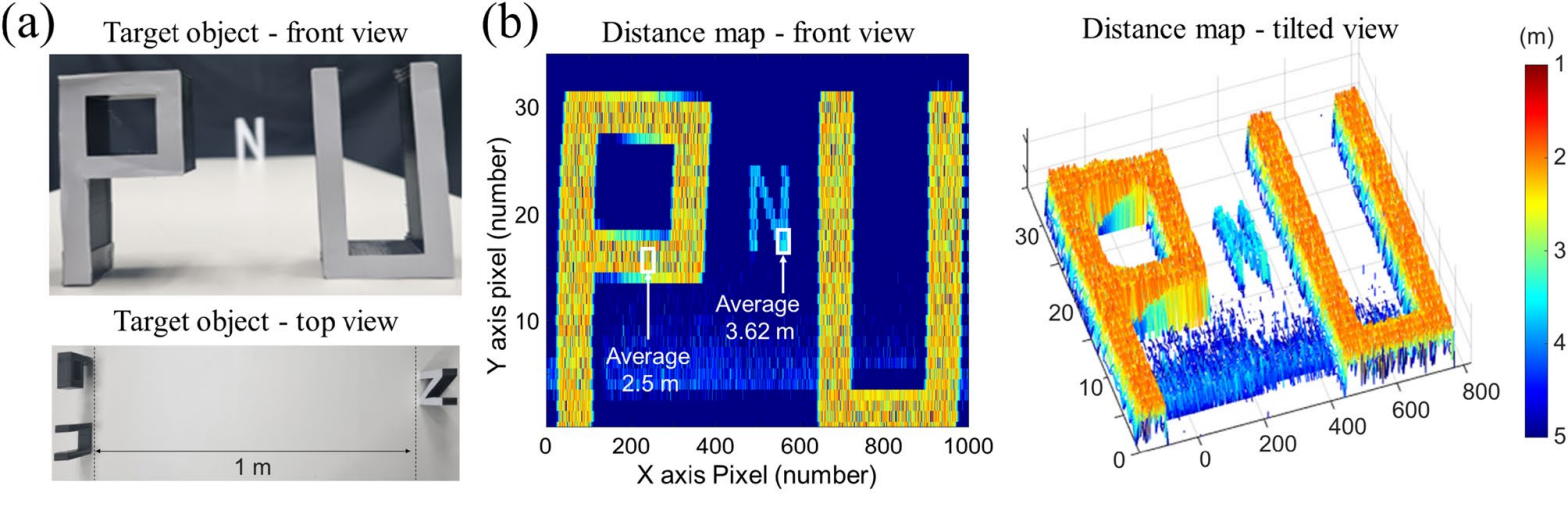
Ranging performance characterization of the multi-spectral LiDAR system. (**a**) Photograph of the target object arrangement showing the front and top view. (**b**) Distance map obtained by the multi-spectral LiDAR system, shown as top view and tilted view. The distance values shown by white arrows are averaged over the respective regions of interest denoted by the white rectangles.

The TDM based multi-spectral LiDAR system permits not only multi-spectral imaging but also distance range mapping simultaneously by measuring the reflected TOF as the delay between a clock trigger pulse and the arrival time of the five-pulse burst. To demonstrate the ranging performance, ‘P’, ‘N’, and ‘U’ letter shaped objects were positioned with the ‘N’ 1 m apart from the other letters in the direction of the LiDAR camera (Fig. 6a) and each object having dimensions of 10 cm height and 5 cm width. The temporal position of the burst is measured by finding the peak of the first pulse in the burst (Fig. 2d), which is more accurate than a simple threshold detector where the relative temporal position of a threshold value can shift with the pulse amplitude. Here, the peak is determined via a rudimentary search for a turning point with linear interpolation for sub-sample numerical accuracy. The search is performed above a certain threshold level in order to exclude background noise from the peak detection and reduce the search range. The (fractional) sample index of the peak within an acquisition record is directly related to the TOF, since each record is hardware timed with the pulse clock trigger.

The processed distance mapping results are shown in Fig. 6b as a front view and as a tilted view. The averaged distance values within the white rectangular regions of interest yield a measured distance between the ‘N’ and the other letters of 1.12 m, which is in good agreement with the actual distance of 1 m. The measured distance error corresponds to a TOF error of about 0.8 ns which is less than one sample at our 1 GS/s sampling rate. The timing and therefore ranging resolution is ultimately limited by the overall electronic timing jitter of our system (clock generation and laser trigger) as well as amplitude noise of the laser sources and the measurement system, which limits the temporal accuracy of the peak detection to about one sample (1 ns).

## Conclusions

In this work, we demonstrated a TDM based multi-spectral LiDAR system, which can provide spatial and spectral information for material identification with both the human eye and machine learning. Our time-domain spectroscopic method not only minimizes optical loss of the system but also enables simultaneous ranging of the target objects. The individual different-wavelength pulses are closely packed in the time domain within a burst, which allows measuring the time-of-flight information over a large distance for ranging the object. Five different nanosecond pulsed lasers in the SWIR range (980 nm, 1060 nm, 1310 nm, 1550 nm, and 1650 nm) were employed to utilize the material-dependent spectral characteristics in the SWIR range for semantic inference classification of different object materials in addition to the object’s shape. Our multi-spectral images show a clear distinction between different materials when mapped into an RGB-color encoded image and are particularly well-suited for systematic classification via the CNN architecture with high accuracy, which makes use of the full spectral information. The proposed technology offers a great potential for the development of compact multi-spectral LiDAR systems to enhance the safety and reliability of autonomous driving.

### Supplementary Information

Supplementary Figure S1.

## Data Availability

The datasets generated and/or analyzed during the current study are available from the corresponding author on reasonable request.
