# Supplementary material for: Time division multiplexing based multi-spectral semantic camera for LiDAR applications
Source: Sci Rep. 2024 May 20;14:11445. doi: 10.1038/s41598-024-62342-2 (PMC11106303; doi:10.1038/s41598-024-62342-2)
Supplement: Supplementary file 1 — Supplementary Figure S1. [file 41598_2024_62342_MOESM1_ESM.docx]

**Supplementary Material**

**Time division multiplexing based multi-spectral semantic camera for LiDAR applications**

Sehyeon Kim^1,†^, Tae-In Jeong^1,†^, San Kim^1^, Eunji Choi^2^, Eunju Yang^2^, Munki Song^2^, Tae Joong Eom^1,2^, Chang-Seok Kim^1,2^*, Alexander Gliserin^1,2^*, Seungchul Kim^1,2^*

† Sehyeon Kim and Tae-In Jeong contributed equally to this work.

^1^ Department of Cogno-Mechatronics Engineering, College of Nanoscience and Nanotechnology, Pusan National University, Busan 46241, Republic of Korea
^2^ Department of Optics and Mechatronics Engineering, College of Nanoscience and Nanotechnology, Pusan National University, Busan, 46241, Republic of Korea

Corresponding Author: Chang-Seok Kim (ckim@pusan.ac.kr), Alexander Gliserin (alex@mpk.or.kr) and Seungchul Kim (s.kim@pusan.ac.kr)

**Keywords**: Time division multiplexing; Lidar; multi-spectral camera; Time of flight

**
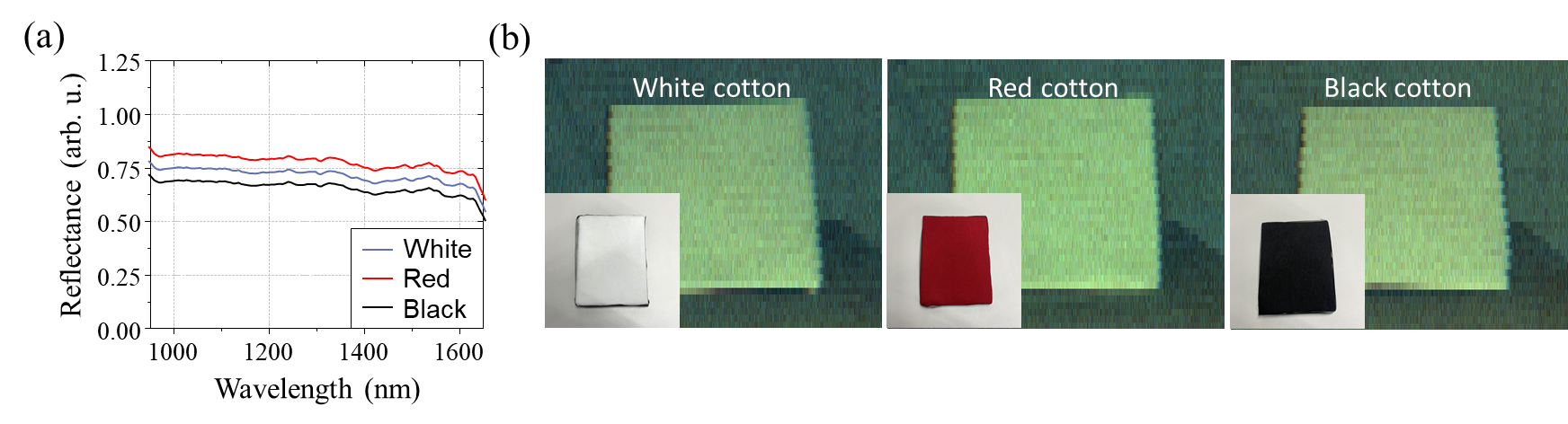
**

Figure S1. **Effect of an object’s color in the visible range on its SWIR reflection spectrum.** (a) Reflection spectrum of red, white, and black colored cotton. (b) Multi-spectral RGB-color encoded images of the colored cotton samples, showing no significant differences in the SWIR range. The inset shows photographs of the objects in the visible range.
